# *VAV2* and *VAV3* as Candidate Disease Genes for Spontaneous Glaucoma in Mice and Humans

**DOI:** 10.1371/journal.pone.0009050

**Published:** 2010-02-04

**Authors:** Keiko Fujikawa, Takeshi Iwata, Kaoru Inoue, Masakazu Akahori, Hanako Kadotani, Masahiro Fukaya, Masahiko Watanabe, Qing Chang, Edward M. Barnett, Wojciech Swat

**Affiliations:** 1 Department of Pathology and Immunology, Hokkaido University Graduate School of Medicine, Sapporo, Japan; 2 National Institute of Sensory Organs, National Hospital Organization Tokyo Medical Center, Tokyo, Japan; 3 Faculty of Health Science, Hokkaido University, Sapporo, Japan; 4 Department of Anatomy, Hokkaido University Graduate School of Medicine, Sapporo, Japan; 5 Department of Ophthalmology and Visual Sciences, Washington University School of Medicine, St. Louis, Missouri, United States of America; 6 Department of Pathology and Immunology, Washington University School of Medicine, St. Louis, Missouri, United States of America; Katholieke Universiteit Leuven, Belgium

## Abstract

**Background:**

Glaucoma is a leading cause of blindness worldwide. Nonetheless, the mechanism of its pathogenesis has not been well-elucidated, particularly at the molecular level, because of insufficient availability of experimental genetic animal models.

**Methodology/Principal Findings:**

Here we demonstrate that deficiency of Vav2 and Vav3, guanine nucleotides exchange factors for Rho guanosine triphosphatases, leads to an ocular phenotype similar to human glaucoma. Vav2/Vav3-deficient mice, and to a lesser degree Vav2-deficient mice, show early onset of iridocorneal angle changes and elevated intraocular pressure, with subsequent selective loss of retinal ganglion cells and optic nerve head cupping, which are the hallmarks of glaucoma. The expression of Vav2 and Vav3 tissues was demonstrated in the iridocorneal angle and retina in both mouse and human eyes. In addition, a genome-wide association study screening glaucoma susceptibility loci using single nucleotide polymorphisms analysis identified *VAV2* and *VAV3* as candidates for associated genes in Japanese open-angle glaucoma patients.

**Conclusions/Significance:**

Vav2/Vav3-deficient mice should serve not only as a useful murine model of spontaneous glaucoma, but may also provide a valuable tool in understanding of the pathogenesis of glaucoma in humans, particularly the determinants of altered aqueous outflow and subsequent elevated intraocular pressure.

## Introduction

The critical importance of elevated intraocular pressure (IOP) in the pathogenesis of glaucomatous optic neuropathy is widely recognized [1], [2]. While compromise of aqueous humor outflow is the key determinant of elevation in IOP [3], [4], the molecular mechanisms underlying changes in the outflow pathway that lead to elevated IOP remain to be elucidated. For this reason, mouse genetic knockout models of spontaneous glaucoma are highly sought after.

The Vav proteins are the best-characterized family of guanine nucleotide exchange factors (GEFs) that activates Rho guanosine triphosphatases (GTPases) in a phosphorylation-dependent manner [5]. Rho GTPases control cell behavior via regulating the specific filamentous actin structures involved in migration, adhesion, and morphogenesis, by acting as binary switches cycling between an inactive (GDP-bound) and active (GTP-bound) state [6]. The three mammalian Vav proteins, Vav1, Vav2, and Vav3, share a Dbl homology domain for their enzymatic activity as GEFs and contain a common structural array characteristic of proteins involved in signal transduction. Regardless of the structural similarity, Vav proteins differ in their tissue distribution. Vav1 is expressed specifically in lymphoid lineage cells, whereas Vav2 and Vav3 are more widely expressed [5], [7]. Genetic approaches using knockout mice have provided valuable information on the function of Vav proteins *in vivo*. Vav proteins are crucial for the development and function of hematopoietic lineage cells such as lymphocytes, neutrophils, natural killer cells, and osteoclasts [8]–[16]. Individual Vav proteins exhibit both redundant and specialized functions. Despite the wide distribution of Vav2 and Vav3 proteins in mouse tissues, little is known about their specific function in non-hematopoietic cells.

While trying to better elucidate the functions of Vav2 and Vav3 in non-hematopoietic cells, we discovered that Vav2/Vav3-deficient mice have a significant ocular phenotype. Specifically, we show that Vav2/Vav3-deficient mice have elevated IOP, which eventually manifests as buphthalmos. Loss of Vav2 and Vav3 expression is associated with changes in the iridocorneal angle, with eventual chronic angle closure. The elevation of IOP in Vav2/Vav3-deficient mice is accompanied by an optic neuropathy characterized by selective loss of retinal ganglion cells (RGCs) and optic nerve head (ONH) excavation and is therefore consistent with glaucoma. In addition, both *VAV2* and *VAV3* are shown to be susceptibility loci by single nucleotide polymorphisms (SNPs) study of Japanese primary open-angle glaucoma patients.

## Results

### Vav2/Vav3-Deficient Mice Develop Buphthalmos

**Figure 1:**
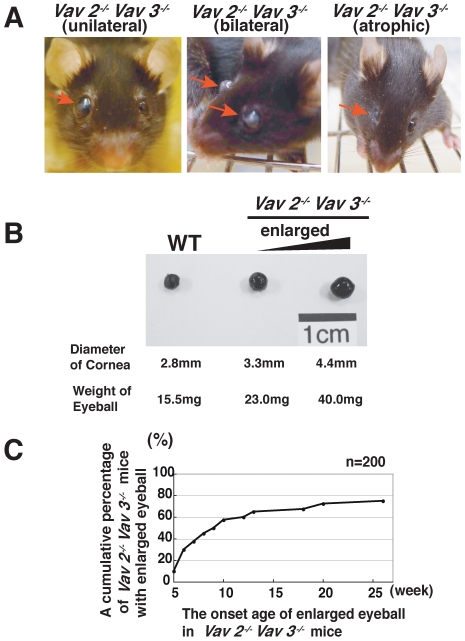
Vav2/Vav3-deficient mice develop buphthalmos. Eyes of Vav2/Vav3-deficient (*Vav2^−/−^Vav3^−/−^*) mice develop buphthalmos between 6 and 12 weeks of age. **A**. Left photo: Representative photo of unilateral enlarged eye in 10-week-old *Vav2^−/−^Vav3^−/−^* mice. Centre photo: Representative photo of bilateral enlarged eyes in 16-week-old *Vav2^−/−^Vav3^−/−^* mice. Right photo: Representative photo of enlarged eye becoming atrophic in 8-week-old *Vav2^−/−^Vav3^−/−^* mice. **B**. Comparison of eye sizes. Left panel: Representative eye of 10-week-old wild-type (WT) mice as a control (n = 20). Cornea diameter is 2.9±0.1 mm. Weight is 15.8±1.1 mg. Centre panel: Representative first-recognized enlarged eye of *Vav2^−/−^Vav3^−/−^* mice (9- to 10-week-old, n = 20). The cornea diameter is 3.3±0.1 mm. Weight is 23.7±4.4 mg. P<0.001. Right panel: Representative moderately enlarged eye of 12-week-old *Vav2* ^−/−^ *Vav3* ^−/−^mice (n = 20). The cornea diameter is 4.2±0.4 mm. Weight is 38.0±4.0 mg. **C**. Age of onset of enlarged eyes up to 25 weeks of age in *Vav2^−/−^Vav3^−/−^* mice (n = 200). The vertical axis is a cumulative percentage of *Vav2^−/−^Vav3^−/−^* mice with enlargement of the eyes.

Eyes of Vav2/Vav3-deficient (*Vav2* ^−/−^ *Vav3* ^−/−^) mice were noted to develop buphthalmos starting between 6 and 12 weeks of age (Figure 1). This enlargement was typically seen unilaterally at first, with frequent bilateral involvement over the next 1–2 months, and continued enlargement until the mice were 6-months old. Eventually, some of the eyes, became atrophic and phthisical in appearance (Figure 1A). In order to confirm our initial observations, we measured the corneal diameters and weights of *Vav2* ^−/−^ *Vav3* ^−/−^ mice eyes and compared them with age-matched wild-type mice eyes (Figure 1B). The examination clearly showed our observations were relevant. We observed 200 *Vav2^−/−^Vav3^−/−^* mice at 6 months of age and almost 75% of them showed the enlarged eyes (Figure 1C). In addition, histological study indicated that there were no abnormal findings in the tissues both around the enlarged eyes such as inflammation, tumor, or hyperplasia, and in the thyroid of the *Vav2^−/−^Vav3^−/−^* mice (data not shown).

### Elevation of Intraocular Pressure of Vav-Deficient Mice

**Figure 2:**
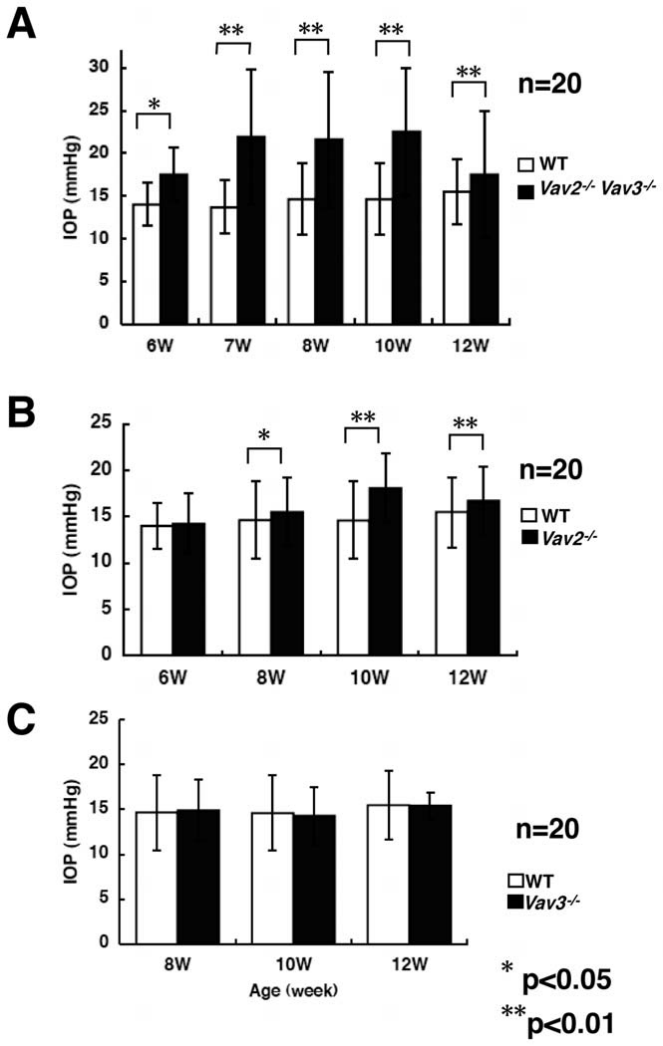
Elevated intraocular pressure of *Vav2^−/−^Vav3^−/−^* and *Vav2^−/−^* mice. The intraocular pressure (IOP) of Vav2/Vav3-deficient (*Vav2* ^−/−^ *Vav3* ^−/−^), Vav2-deficient (*Vav2* ^−/−^), and Vav3-deficient (*Vav3* ^−/−^) mice were measured between 10–12 AM. At the indicated ages, twenty mice were examined, respectively. For the IOP measurement of each Vav-deficient mouse, IOP of an age-matched wild-type (WT) mouse was also measured under the same conditions. We confirmed that these results were reproducible with four independent examinations. **A**. IOPs of *Vav2* ^−/−^ *Vav3* ^−/−^ mice were dramatically elevated at 6 weeks of age. **B**. *Vav2* ^−/−^ mice also showed elevated IOP from around 8 weeks of age. **C**. *Vav3* ^−/−^ mice have normal range of IOP at any age. Error bars represent S.D. *P<0.05, **P<0.01 versus WT mice.

As we observed the development of buphthalmos, we assessed for elevated IOP in *Vav2* ^−/−^ *Vav3* ^−/−^, Vav2-deficient (*Vav2* ^−/−^), and Vav3-deficient (*Vav3* ^−/−^) mice. IOP was measured using a rodent tonometer (Tonolab) starting at 4 weeks post-natal and were compared with age-matched wild-type C57BL/6 mice. Reliable measurement of IOP before 4 weeks of age was not possible. At 6 weeks of age, *Vav2^−/−^Vav3^−/−^* mice first showed increased IOP (18.2±3.1 vs. 14.0±2.4 mmHg, p<0.05), with further increases out to 10 weeks of age (22.5±7.4 vs. 14.6±4.2 mmHg, p<0.01) (Figure 2A). IOP measurements in *Vav2^−/−^Vav3^−/−^* mice ranged from 11–40 mmHg between 7 weeks and 16 weeks of age. There was a statistically significant difference in IOP between the *Vav2^−/−^Vav3^−/−^* and wild-type mice at all time points demonstrated. The phenotype of littermate wild type mice was identical to that of the “inbred” C57BL/6 strain (Figure S1).

In *Vav2^−/−^* mice, elevated IOP was first detected at 7 weeks of age. The IOP for *Vav2^−/−^* mice was found to be increased at 8 weeks of age compared to wild-type mice (15.5±3.7 vs. 14.0±4.2 mmHg, p<0.05)(Figure 2B). The IOP of *Vav2* ^−/−^ mice showed further increases at 10 weeks of age (18.1±3.7 vs. 14.6±4.2 mmHg, p<0.01) and remained significantly higher at 12 weeks. In contrast, the IOP of *Vav3^−/−^* mice did not differ significantly from wild-type mice between 8 and 12 weeks (Figure 2C). The phenotype of littermate wild type mice was identical to that of inbred strain “C57BL/6”. We also demonstrated that the phenotype of Vav2 and Vav3 heterozygous littermate mice (*Vav2* ^+/−^, and *Vav3* ^+/−^) were same as that of wild type (Figure S1).

### Retinal Ganglion Cell Loss and Optic Nerve Head Changes in Vav2/Vav3-Deficient Mice

**Figure 3:**
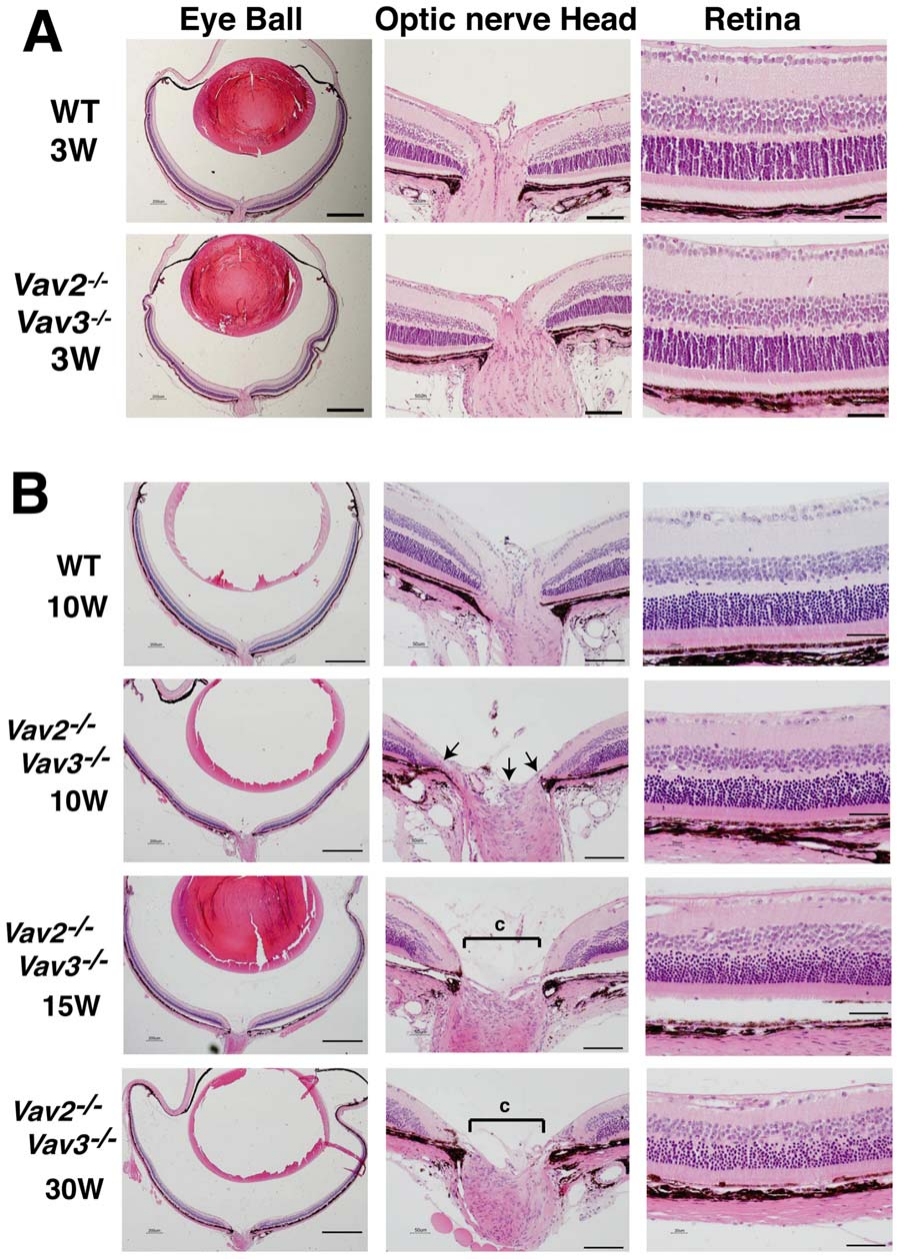
Optic nerve head degeneration and decrease in RGCs observed in *Vav2^−/−^Vav3^−/−^* mice with elevated IOP. Light-microscopic histological examination is conducted to evaluate retinal neuropathy in Vav2/Vav3-deficient (*Vav2^−/−^Vav3^−/−^*) mice. **A**. At the age of 3 weeks, *Vav2^−/−^Vav3^−/−^* mice exhibited impairment of angle status, but no abnormal findings of Optic nerve head degeneration (ONH) or retinal ganglion cells (RGCs) in the retinas. Scale bars, from left to right side: 500 µm, 100 µm, and 50 µm. **B**. After elevation of IOP, compared to control wild-type (WT) mice in the upper panel, ONH in 10-, 15-, and 30-week-old *Vav2* ^−/−^ *Vav3* ^−/−^ mice present so-called capping (shown in c) and thin retinal neural layers (indicated by arrows in the photos). In those retinas, RGCs are decreased. Scale bars, from left to right side: 500 µm, 100 µm, and 50 µm. Sections are representative from 6–12 samples.

We next examined whether Vav2/Vav3-deficient (*Vav2* ^−/−^ *Vav3* ^−/−^) mice showed changes in the retinal ganglion cell (RGC) layer and optic nerve head (ONH). At 3 weeks of age, *Vav2* ^−/−^ *Vav3* ^−/−^ mice did not show any histological difference in the ONH or the number of RGCs compared to that of age-matched wild-type mice (Figure 3A). At 10 weeks of age, following several weeks of IOP elevation, early signs of ONH cupping and cell body loss in the RGC layer were apparent in *Vav2^−/−^Vav3^−/−^* mice (Figure 3B). At 15 and 30 weeks of age, *Vav2* ^−/−^ *Vav3* ^−/−^ mice showed further evidence of ONH cupping and RGC loss in the context of an otherwise normal retinal architecture. These findings are consistent with a selective loss of RGCs with corresponding changes in the ONH, which are the hallmarks of glaucoma.

### Iridocorneal Angle Histopathology in Vav-Deficient Mice

**Figure 4:**
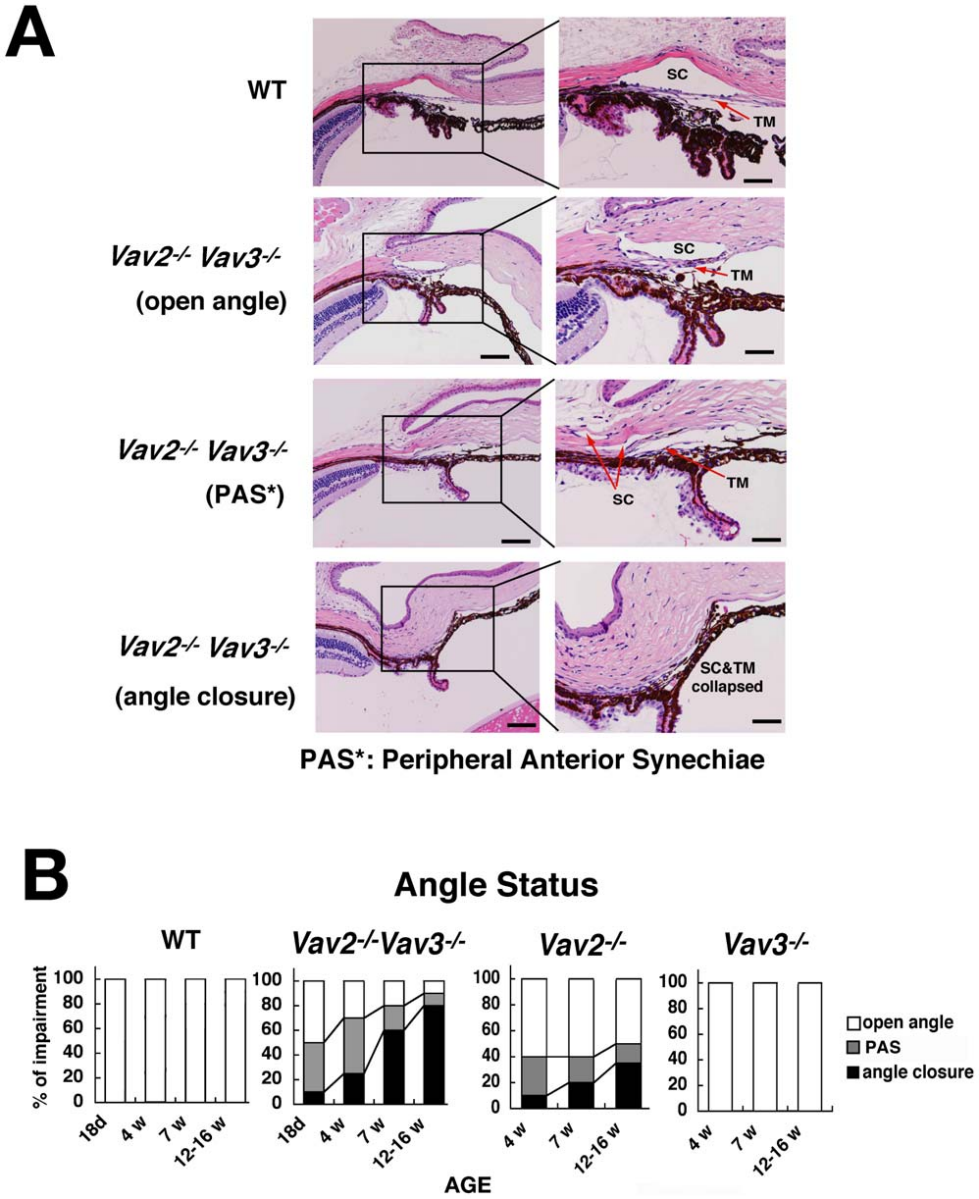
Characterization of progressive iridocorneal angle closures in *Vav2^−/−^Vav3* ^−/−^ and *Vav2* ^−/−^ mice. The aqueous humor outflow facility, trabecular meshwork (TM) and Schlemm's canal (SC) (iridocornial angle) in Vav2/Vav3-deficient (*Vav2^−/−^Vav3* ^−/−^) mice are evaluated in histological manner. Vav2-deficient (*Vav2* ^−/−^) mice also have the same changes, but of lower severity. **A**. Representative photos of normal TM and SC histology of 12-week-old wild-type (WT) mice as a control. Representative photos of normal open angle, peripheral anterior synechiae (PAS) in 12-week-old *Vav2^−/−^Vav3* ^−/−^ mice, and angle closure status in 12-week-old *Vav2^−/−^Vav3* ^−/−^ mice. Sections used here are all representative from 20 samples. Scale bars: left photos, 200 µm; right photos, 100 µm. **B**. Changes of angle status appear at the early ages. We classify angle status of *Vav2^−/−^Vav3* ^−/−^, *Vav2* ^−/−^, and *Vav3* ^−/−^mice into open angle, PAS, and angle closure by histological evaluation. We find the changes of angle status at the early ages, such as in 18-day-old *Vav2^−/−^Vav3^−/−^ mice* (n = 20) and in 4-week-old of *Vav2^−/−^Vav3^−/−^ mice* (n = 20). We took four (*Vav2* ^−/−^ *Vav3* ^−/−^) and three (*Vav2* ^−/−^, *Vav3* ^−/−^) different age groups, with 20 mice examined, respectively.

As histopathological examination of globes from mice with buphthalmos frequently demonstrated angle closure, we compared the iridocorneal angle histology of 20 Vav2/Vav3-deficient (*Vav2* ^−/−^ *Vav3* ^−/−^) mice with wild-type mice at both 7 and 12 weeks of age. Angles were classified as either being completely open, displaying evidence of partial occlusion of the trabecular meshwork (TM) as manifest by peripheral anterior synechiae (PAS), or being completely closed (total occlusion of the trabecular meshwork)(Figure 4A). Over half of the Vav2/Vav3-deficient mice already showed evidence of angle closure by 7 weeks of age, increasing to nearly 80% in 12-week-old mice (Figure 4B).

We also examined the correlation between elevated IOP and angle changes in 7-week-old *Vav2* ^−/−^ *Vav3* ^−/−^ mice respectively (n = 20) (Figure S2). The mean and standard deviation of IOP in 7-week-old wild-type mice (n = 18) were 13.7±3.12 mmHg respectively. The 95th percentile of those IOPs using a normal curve was 18.8 mmHg. So that IOP over 18.8 mmHg was regarded as elevated IOP. *Vav2* ^−/−^ *Vav3* ^−/−^ mice with elevated IOP showed evidence of angle closure by histological analysis, while *Vav2* ^−/−^ *Vav3* ^−/−^ mice with non-elevated IOP displayed either open angles or evidence of early angle closure (PAS) and angle closure.

In addition, to characterize the progression of angle changes, two additional time points were added to this analysis of the iridocorneal angle −18 days and 4 weeks of age (n = 20 each). While at 18 days of age nearly half of the eyes demonstrated open angles, a large percentage already showed evidence of PAS (Figure 4B). By 4 weeks of age, *Vav2* ^−/−^ *Vav3* ^−/−^ mice showed increasing frequencies of both PAS and angle closure. Taken as a whole, the data showed a gradual progression from open angles to PAS formation to closed angle from 18 days to 12 weeks.

The iridocorneal angles of Vav2-deficient (*Vav2* ^−/−^) and Vav3-deficient (*Vav3* ^−/−^) mice were examined histologically and graded in a similar manner. The iridocorneal angles of *Vav2^−/−^* mice also demonstrated evidence of progressive angle closure, but to a lesser extent as compared with *Vav2^−/−^Vav3^−/−^* mice (Figure 4B). *Vav3* ^−/−^ mice had normal appearing open angles without evidence of PAS formation or angle closure (Figure 4B).

In order to better investigate the status of iridocorneal angles in *Vav2* ^−/−^ *Vav3* ^−/−^ mice, we stained for myocilin as a marker for TM cells, as myocilin is strongly expressed in TM cells [17]. We examined 7-week-old *Vav2* ^−/−^ *Vav3* ^−/−^ mice with non-elevated IOP who had either open angles or who displayed evidence of angle closure. As shown in Figure S3, myocilin was not detected in the iridocorneal angle of *Vav2* ^−/−^ *Vav3* ^−/−^ mice with angle closure, but was seen in mice with open angles similar to those of wild-type mice.

### Effects of Ocular Hypotensives in Vav2/Vav3 -Deficient Mice

We next tested the efficacy of ocular hypotensives used for human glaucoma in *Vav2* ^−/−^ *Vav3* ^−/−^ mice with elevated IOP (Figure S4). The elevated IOP of 7-week-old *Vav2* ^−/−^ *Vav3* ^−/−^ mice was dramatically reduced by ocular hypotensives used in humans, such as latanoprost, a prostaglandin analogue (Figure S4A). We also tested the IOP-lowering effect in *Vav2* ^−/−^ *Vav3* ^−/−^ mice by two other ocular hypotensives, dorzolamide and timolol, whose mechanisms of action differ from that of latanoprost [18]–[20], being aqueous suppressants (Figure S4B). Furthermore, we tested Y-27632, a Rho-associated protein kinase inhibitor, that has been reported to cause a reduction in IOP presumably by altering cellular behavior of TM cells [21]–[23]. Y-27632 showed no effect of lowering IOPs against *Vav2* ^−/−^ *Vav3* ^−/−^ mice, while it lowered the IOP significantly in age-matched wild-type mice (Figure S4C).

### Expression of Vav2 and Vav3 in Mouse and Human Eyes

**Figure 5:**
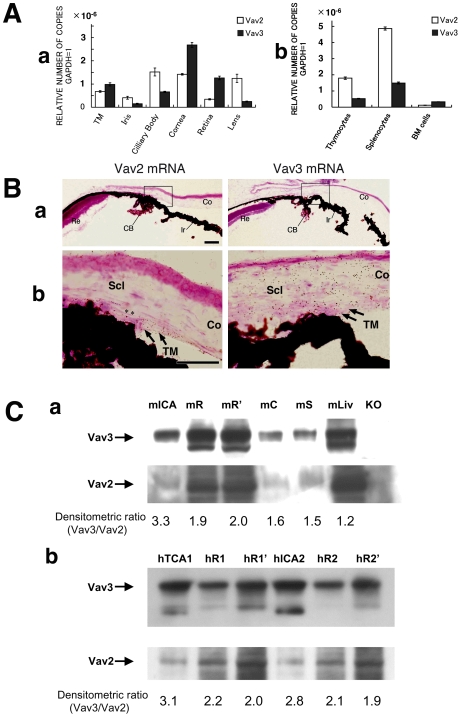
Vav2 and Vav3 expression in mouse and human eyes. **A**. Quantitative real time PCR analysis is performed for Vav2 and Vav3 mRNA expression study. The vertical axis is the copy number of Vav2 or Vav3 mRNA when that of mGAPDH is taken as 1. The assay method is absolute quantification (standard curve). **a.** Both Vav2 and Vav3 mRNA are expressed in all tissues of the murine eyes including the trabecular meshwork (TM), cornea, sclera, and retina. **b.** Vav2 and Vav3 mRNA expression level of murine immune cells. The levels of Vav2 and Vav3 expression in eye tissues are the same as those of the immune cells where Vav2 and Vav3 play the critical role. **B. a.** In situ hybridization analysis of emulsion-dipped sections display the distribution of Vav2 and Vav3 mRNA in the anterior chamber. The localization of Vav2 and Vav3 mRNA in trabecular meshwork(TM), ciliary body (CB), cornia(CO), iris(Ir), sclera (Scl) and retina(Re) by in situ hybridization. **b.** Vav2 and Vav3 mRNA expression are both detected in iridocorneal angle, such as TM (indicated by arrows in the photos). Scale bars, 50 µm. **C.** Expression of Vav2 and Vav3 proteins in mouse (**a**) and human (**b**) eyes. Vav2 and Vav3 proteins were detected in mouse or human ocular extracts (from two independent postmortem eye globe samples; at death age of 58 (1) and 87 (2)) by western blotting. Densitometric ratios (Vav3/Vav2) were shown under the blotting panels. mICA: mouse iridocorneal angle tissues, mR: mouse retina, mR': 3-fold increased loading mouse retina, mC: mouse cornea, mS: mouse sclera, mLiv: normal mouse liver(positive control), KO: Vav2/Vav3-deficient mouse as a negative control, hICA: human iridocorneal angle tissue,hR1: human retina 1, hR1': human retina1' (3-fold loading).

In order to understand the pathogenesis of the Vav2/Vav3-deficient eye phenotype, we examined the mRNA and protein expression patterns of Vav2 and Vav3 in the eye (Figure 5). Quantitative real-time PCR revealed that Vav2 and Vav3 mRNA are expressed in TM, cornea, retina, lens, iris, and ciliary body in the mouse eye (Figure 5A). Vav3 mRNA was more abundantly expressed than that of Vav2 in the TM and the retina. Gene expression levels of both Vav2 and Vav3 in the eye were comparable to levels found in immune cells where Vavs play a critical role [5], [7]–[16]. Next, the Vav2 and Vav3 mRNA localization in mouse eye was examined by in situ hybridization (ISH) analysis (Figure 5B). Both Vav2 and Vav3 oligo probes (antisense), we used here, have been examined the specificities before and proved to have its specificity. As negative controls for these experiments, we used sense probes of Vav2 and Vav3, respectively, which showed no detectable signal (Figure S5). Both genes expression were widely distributed in the ocular tissues including the iridocorneal angle, retina, cornea, and sclera. The co- localization of Vav2 and Vav3 mRNA expression in iridocorneal angle, such as TM, was confirmed by ISH. Also, we assessed Vav2 and Vav3 protein expression by immunoblotting in both mouse and human eyes (Figure 5C). In mouse eyes, expression of both Vav2 and Vav3 was demonstrated in several ocular tissues including the iridocorneal angle, retina, cornea, and sclera. Both Vav2 and Vav3 proteins were also expressed in human retina and iridocorneal angle. The migrated bands were absent in the liver extracts of the *Vav2* ^−/−^ *Vav3* ^−/−^ mice. Results of densitometric ratio (Vav3/Vav2) from normalized protein loading in each lane revealed that Vav3 was more abundantly expressed than Vav2 in the iridocorneal angle tissues of both mouse and human eyes and also in the retina.

### Single Nucleotide Polymorphisms in Japanese Primary Open-Angle Glaucoma Patients

We observed Vav2 and Vav3 proteins expression in the tissues of human iridocorneal angle and retina. In order to investigate the relevant association of *VAV2* and *VAV3* in human glaucoma patients, we carried out a genome-wide association study using the Affymetrix GeneChip Human Mapping 500 K Array Set. We examined Japanese primary open–angle glaucoma (POAG) cases and age-matched non-glaucoma controls. Both *VAV2* and *VAV3* loci in Japanese POAG patients showed SNPs against the non-glaucoma controls for dbSNPs rs2156323 and rs2801219, respectively. We reported the most extreme (Table 1). Both were intronic SNPs, SNP rs2156323 lying in intron3 of *VAV2* and SNP rs2801219 lying in intron1 of *VAV3*. *VAV2* SNP rs2156323 in particular indicated significant association with Japanese POAG, including a 5.65 heterozygote odds ratio (95% confidence interval (CI): 1.99–16.0), 4.34 heterozygote relative risk (95% CI: 1.72–10.44) and 4.38×10^−4^ genotypic *P* value with respect to risk allele A.

**Table 1:** Vav2, Vav3, Vav1 association study for POAG using the Affymetrix GeneChip.

Gene	*VAV2*	*VAV3*	*VAV1*
SNP ID	rs2156323	rs2801219	rs2617815
Chromosome Location	9q34.1	1p13.3	19p13.2
Position	133750375	108214454	6746147
Genotypic *P* value	4.38×10^−4^	5.42×10^−4^	4.41×10^−2^
Allele	AG	AC	AG
Risk allele	A	C	G
Minor allele	A	C	G
Heterozygote odds ratio (95%CI)	5.65 (1.99–16.0)	2.03 (1.01–4.09)	1.04 (0.52–2.08)
Heterozygote relative risk (95%CI)	4.34 (1.72–10.44)	1.31 (1.00–1.75)	1.01 (0.82–1.23)
Homozygote odds ratio	Not Available	Not Available	Not Available
Exon Intron	*VAV2* Intron3	*VAV3* Intron1	*VAV1* Intron1
SNP type	iSNP*1	iSNP	iSNP

* 1: intronic S.

**Figure 6:**
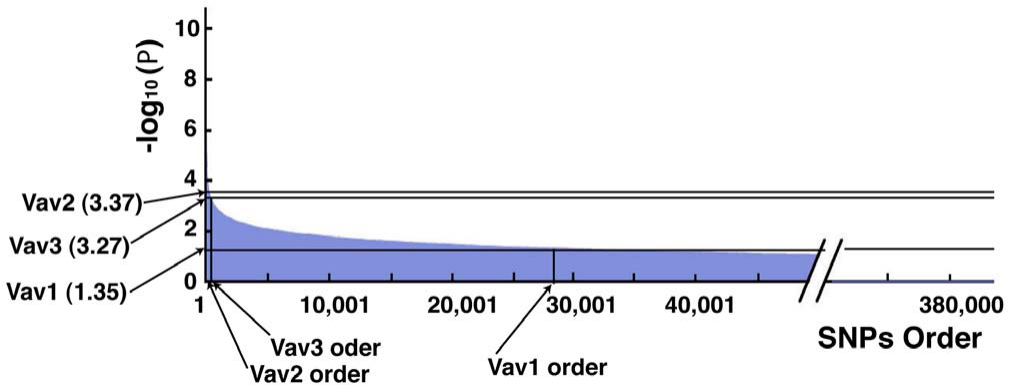
*VAV2* and *VAV3* genome-wide SNPs high ranking of *P*-value scores. Genome-wide ranking orders of *P*-value indicate that *VAV2* and *VAV3* are strongly susceptible genes with Japanese POAG cases. Clinically diagnosed Japanese POAG 100 cases and non-glaucoma age-matched 100 controls are examined for this study. The analysed SNPs number is about 380,000 by the Affymetrix GeneChip 500 K Mapping Array Set. The SNPs data under the 85% call rate, under 0.001 Hardy-Weinberg equilibrium (HWE), and under 5% minor allele frequencies are excluded. Allelic frequency χ2 test and genotypic frequency χ2 test are calculated respectively. The vertical axis is −log10 (*P*) and the horizontal axis is SNPs order which showed high scores from left to right. The Upper graph is alleric *P*-values distribution of *VAV2* analysis and the lower graph is genotypic *P*-values for *VAV3* and *VAV1* study. *VAV2* is located at high position in rank and *VAV3* also located at high position in rank. *VAV1* shows no association for POAG cases here.

Judging from alleric *P*-values distribution for detecting *VAV2* ranking and genotypic *P*- values distribution for *VAV3* ranking, we observed that *VAV2* and *VAV3* showed high scores (−log_10_(*P*)) among approximately 380,000 SNPs analyzed in this study (Figure 6). On the contrary, *VAV1* showed no association with the POAG. These data strongly suggest that *VAV2* and *VAV3* genes are susceptibility loci in Japanese POAG.

## Discussion

To our knowledge, this is the first report of a spontaneous glaucoma phenotype in Vav2 (*Vav2^−/−^*) or Vav2/Vav3-deficient (*Vav2* ^−/−^ *Vav3* ^−/−^) mice. Vav2/Vav3-deficiency is associated with progressive iridocorneal angle changes and elevation of IOP in mice. Subsequent selective loss of RGCs and progressive ONH cupping are associated with this elevated IOP, as has previously been demonstrated in other rodent models of glaucoma [24]. The finding that Vav2-deficiency alone results in a glaucoma phenotype suggests that the absence of Vav2 plays a critical role in the development of this phenotype. Despite our finding that Vav3-deficiency did not result in either iridocorneal angle changes or elevated IOP, the more severe glaucomatous phenotype demonstrated in *Vav2^−/−^Vav3^−/−^* mice as compared with *Vav2^−/−^* mice is consistent with an additive effect.

A number of induced glaucoma models have been established in rats and mice [24]. Each model has advantages and disadvantages, related to factors such as the ease of inducing elevated IOP, the magnitude, duration and variability of elevated IOP, and secondary effects on the eye. Due to the ease of genetic manipulation, mouse models are becoming increasingly popular over those in rats. Despite the lack of a lamina cribosa as found in human eyes, the mouse is a good genetic model to study the pathogenesis of human glaucoma as aqueous physiology and anterior segment anatomy are similar to that found in humans [25].

Other spontaneous models of glaucoma have been described in mice, most notably in DBA/2J mice. The pigmentary glaucoma phenotype demonstrated in the DBA/2J mice has been extensively studied at genetic, clinical, morphological and pathological levels [26]–[29]. A limitation of this model is that the elevated IOP phenotype is not primary but secondary due to the systemic pigment dispersion syndrome with the associated mutations in the *Gpnmb* and *Tyrp1* loci [26]–[30]. In these mice, recessive mutations in these 2 genes are associated with iris degeneration characterized by iris stromal atrophy and pigment dispersion with subsequent reduced outflow facility secondary to pigment and cell debris. Therefore, it is difficult to tie-in the identified mutations to the pathogenesis of any primary form of human glaucoma.

The Vav2/Vav3-deficient mouse has several characteristics which make it particularly useful as an animal glaucoma model. The elevated IOP occurs spontaneously in these genetically manipulated mice and does not require the ocular manipulation necessary in induced models. The frequency of the ocular phenotype is high and onset occurs at a relatively young age. In addition, ocular hypotensives commonly used to treat human glaucoma show efficacy in lowering IOP in this model. The most significant advantage of this mouse glaucoma model is that the deleted genes, Vav2 and Vav3, are well-focused targets that have been studied over 20 years providing a useful starting point for further investigation of the potential molecular mechanisms underlying this phenotype.

Several aspects of this model of spontaneous glaucoma will require further study and clarification, although we speculated from our histological results and the correlation between elevated IOP and angle status changes that anatomic angle closure is the possible mechanism for elevated IOP in this model. While progressive angle closure may be the etiology prior to elevated IOP in mice lacking Vav2 and Vav3 function, it may alternatively be a subsequent change related to other alterations in angle structures which might also affect aqueous humor outflow. In addition, since the expression of Vav2 and Vav3 was detected in ocular tissues other than those comprising the iridocorneal angle, it will be necessary in future studies to consider how their deficiency in these tissues might have potentially contributed to the spontaneous glaucoma phenotype in any way.

While so far there are several reports of glaucoma associated candidate genes based on the single nucleotide polymorphisms (SNPs) study in the Japanese population [31]–[36], our data first suggest that *VAV2* and *VAV3* are susceptibility loci in Japanese primary open–angle glaucoma (POAG) cases. In addition, so far we could not find the report of non-Japanese glaucoma association case study that demonstrated *VAV2* and/or *VAV3* as candidate gene loci for glaucoma [37]–[43]. They demonstrated glaucoma associated candidate genes study with SNPs analysis focusing on the other specific target genes, although we are interested in the *VAV2* and/or *VAV3* glaucoma association study using the different populations. This work would be important investigation to be done.

Although our current findings do not address the molecular mechanisms underlying glaucoma phenotypes, it is interesting to consider possible mechanisms based on what is currently known about Vav protein function. The TM has been regarded as a key determinant of IOP and has been implicated as the major site of increased resistance to aqueous outflow which occurs in human glaucoma [44], [45]. Recent findings indicate that signals emanating from integrins, key regulators of the actin cytoskeleton in trabecular meshwork cells, may be involved in control of outflow facility and Rho GTPases would be important downstream effectors of integrin-mediated actin cytoskeletal dynamics [4], [46]–[48]. Considering the Vavs function as GEF, dysregulation of Rho is one possible mechanism by which pathology in the iridocorneal angle might result and is one that deserves further study.

In summary, we had demonstrated that Vav2/Vav3-deicient mice develop a spontaneous glaucoma phenotype. In addition, our data first suggest that *VAV2* and *VAV3* are susceptibility loci in Japanese primary open-angle glaucoma (POAG) cases. We believe that Vav2/Vav3-deficient mice will serve not only as a useful murine model of spontaneous glaucoma, but may also provide a valuable tool in understanding of the pathogenesis of glaucoma in humans, particularly the determinants of altered aqueous outflow and elevated IOP.

## Materials and Methods

### Mice

*Vav3* ^−/−^, *Vav2* ^−/−^ and *Vav2* ^−/−^ *Vav3* ^−/−^ mice were described previously [15]. Mice were backcrossed at least 9 times with C57BL/6 mice (Clea Japan, Tokyo, Japan) to have the C57BL/6 background. All mice used in these experiments were bred and maintained in the SPF Facility of Hokkaido University Graduate School of Medicine in a 12-hour light-dark cycle. All mice experiments were approved by the Animal Ethics Committee of Hokkaido University Graduate School of Medicine and were conducted in accordance with the ARVO Statement for the Use of Animals in Ophthalmic and Vision Research.

### Tissue Preparation and Histology

Eyes were quickly enucleated from each age group of knock-out mice and C57BL/6 wild-type control mice after deep anesthesia with pentobarbital sodium solution, then immediately fixed with solution of 2.5% glutaraldehyde (TAAB, EM Grade) in 10% formalin neutral buffer-methanol solution deodorized for anterior chamber study, or fixed with Davidson' solution for retinal analysis for 12 hours. Following this, the eyes were embedded in paraffin and dissected sagittally using a microtome into 5 µm sections. After deparaffinization and rehydration, the sections were stained with hematoxylin and eosin (Sigma).

### Immunohistochemistry

The eyes were sectioned at 5 µm thickness along the vertical meridian through the optic nerve head. After deparaffinization and rehydration, the tissue sections were incubated with blocking solution containing 1% BSA in PBS for 1 hour. This was followed by 1 hour incubation with rabbit polyclonal antibody to myocilin at 1∶200 in blocking solution as first antibody for 1 hour at room temperature. Anti-rabbit IgG conjugated with Alexa 488 (Molecular Probes, Eugene, OR) at 1∶400 in PBS containing 0.1% Tween 20 was used as secondary antibody for 1 hour at room temperature. The stained tissues were examined using confocal fluorescence laser microscope (Radius 2000, Bio-Rad, Hercules, CA). For negative control of the immunohistochemical staining, the sections were incubated with blocking solution without primary antibody (data not shown).

### Real Time PCR

Each tissue was freshly taken from SPF level C57BL/6 mice and immediately used for generating RNA by TRIzol reagent (Invitrogen). Templates for real time PCR were made by Cloned AMV Reverse Transcriptase (Invitrogen). Probes of mVav2 and mVav3 were TaqMan probes (Vav2: Mm00437287_m1, Vav3: Mm00445082_m1) purchased from Applied Biosystems (Foster city, CA). The standard curves were constructed by mVav2, mVav3 inserted plasmids, normalized by mGAPDH (Product Code: 4352339E, Applied Biosystems). All the PCR studies were performed by Applied Biosystems 7500 Real Time PCR System following the manufacturer's recommended procedures. The assay method was absolute quantification (standard curve).

### In Situ Hybridization

The detailed procedure was described as previously [49]. Briefly, to detect mRNAs for Vav2 and Vav3, specific antisense oligonucleotide probes were synthesizedas follows:(2275–2319;45mers)5′-AGCTGGAGACCGGCTTGAGGCC CTGCTGGTGGTTCGCTCCCGAGA-3′ for Vav2 mRNA (GenBank accession No. NM_009500) and (2346–2302;45mers)5′–GTTGCCTGTTCTATTACCCCTCTG TCCAGCTGGCTGTTCTGGCTC-3′ for Vav3 mRNA (accession No. NM_020505). Oligonucleotide probes were labeled with [33P] dATP using terminal deoxyribonucleotidyl transferase (Invitrogen, Carlsbad, CA). Under deep pentobarbital anesthesia, the eyeballs were freshly obtained from Adult C57BL/6J mice. Fresh frozen sections (20 µm thickness) were cut with a cryostat (CM1900, Leica, Nussloch,Germany) and mounted on glass slides precoated with 3-aminopropyltriethoxysilane. Sections were exposed to Nuclear Track emulsion (NTB-2, Kodak) for 5 weeks. Emulsion-dipped sections were stained with methyl green pyronine solution. The specificity of the hybridizing signals was verified by the disappearance of signals when hybridization was carried out with sense probes.

### Western Blotting

Mouse Ocular Tissue Dissection: 8-week male C57BL/6J mice (Jackson Laboratory, ME) were used for ocular tissue samples. The animals were euthanized by carbon dioxide inhalation in an induction chamber. The globes were promptly enucleated after euthanization and washed in ice-cold PBS. Ocular tissues were microscopically dissected. Dissection of Postmortem Human Eye Globes: Human eyes without previous eye diseases including glaucoma were acquired from a local eye bank (Heartland Lions Eye Banks; Columbia, MO) within 6 hours post-mortem. Dissected mouse and postmortem human ocular tissues were lysed in a tissue extraction buffer (BioChain, CA). The concentration of protein supernatants was determined by a protein assay kit (Bio-Rad, CA). Rabbit polyclonal anti-mouse Vav2 (1∶1000) (Santa Cruz Biotechnology, CA), monoclonal anti-human Vav2 (1∶2000) (Cell Signaling Technology, MA), polyclonal anti-mouse and anti-human Vav3 (1∶3000 for each) (Millipore, CA) antibodies were used for detection.

### Intraocular Pressure (IOP) Measurement

IOP was measured using the TonoLab rebound tonometer for rodents (Tiolat i-care, Finland) according to the manufacturer's recommended procedures. All IOP measurements were performed between 10 AM and noon in conscious condition. Mice were gently restrained first by hand and placed on a soft towel bed on the desk and usually appeared calm and comfortable. These data were confirmed to be reproducible by three additional different independent studies (n = 20).

### Evaluation of Eye Drop Medications for High Intra-Ocular Pressure of Vav2Vav3-Deficient Mice

*Vav2* ^−/−^ *Vav3* ^−/−^ mice were housed in SPF barrier facility in standard lighting conditions (12-hour light-dark cycle). The 7–9 week after birth mice were used for the experiment. Four independent experiments were carried out to confirm the results reproducible.

### Preparation and Application of Ophthalmic Solution

Latanoprost was purchased from Cayman Chemical Co. (Ann Arbor, MI) and dissolved in its vehicle solution (0.02% benzalkonium chloride, 0.5% monosodium phosphate monohydrate, 0.6% disodium hydrogen phosphate dihydrate and 0.4% sodium chloride). With a micropipette, 3 µl of PG analogue (latanoprost; prostaglandin F2α) solution or vehicle was randomly applied to the eyes of *Vav2* ^−/−^ *Vav3* ^−/−^ mice. Before administration, IOP was measured with the tonometer from 10–12 AM and then the PG analogue 0.005% 3 µl or vehicle solution was applied in a masked manner. Evaluation of IOP-lowering effect was performed by measuring the IOP with the tonometer at 3 hours after drug instillation also in a masked manner. Furthermore, two different mechanistic medications, 3 µl of timolol maleate (0.5%, Merck, Whitehouse Station, NJ) or 3 µl of dorzolamide hydrochloride (1%, Trusopt; Merck), was also tested, respectively, after measuring the IOP under the same conditions as those of the Latanoprost application. Evaluation of IOP-lowering effects was performed by measuring the IOP with tonometer at 2 hours after drug instillation under blinded test protocols. Y-27632 was purchased from Carbiochem (La Jolla, CA) and dissolved in its vehicle solution (phosphate buffered saline). Y-27632 (1 mM) or vehicle solution was administered to the central cornea as a 3 µl drop by pipetting in a masked manner. Evaluation of IOP-lowering effect was performed by measuring the IOP with the tonometer at 1 hour after drug instillation.

### Statistical Analysis of IOPs

Data are reported as means ± S.D. Two-tailed Student's t-test was used to compare between two groups of results. Differences between any two groups were regarded as significant when P<0.01(**) or P<0.05 (*).

### Disease Associated Genome-Wide Analysis

One hundred clinically-diagnosed cases (male 46; female 54) with primary open-angle glaucoma over 30 years of age (mean age, 71.60 years; SD, 9.33 years) and non-glaucoma age-matched controls (mean age, 66.71 years; SD, 12.00 years) in a Japanese population were examined for this study. Informed consent was obtained from all participants, and the procedures used conformed to the tenets of the Declaration of Helsinki. Genomic DNAs were isolated from the peripheral blood of the POAG cases and age-matched controls for genotyping analysis. Genotyping was performed using the Affymetrix GeneChip Human Mapping 500 K Array Set (Affymetrix Services Laboratory, California). We omitted the SNP data under an 85% call rate, under 0.001 Hardy-Weinberg equilibrium (HWE), and under 5% minor allele frequency. Data analysis was performed using the LaboServer System (World Fusion, Tokyo Japan). An allelic frequency χ^2^ test and genotypic frequency χ^2^ test were calculated, respectively with respect to risk allele. The Odds ratio was calculated in three manners such as per allele odds ratio, heterozygote odds ratio, and homozygote odds ratio. Relative risk was also calculated, the same as for the odds ratio. The most significant SNPs were chosen in this report to evaluate the association of *VAV2*, *VAV3*, and *VAV1* in the cases.

## Supporting Information

Figure S1 The comparison of intraocular pressures in age matched wild-type inbred C57BL/6 mice, wild-type littermate controls, and Vav2 and Vav3 heterozygous mice (Vav2^+/−^, and Vav3^+/−^). Intraocular pressures (IOPs) were measured using the TonoLab rebound tonometer for rodents from 6-week to 12-week, as described in the Methods. The phenotype of littermate wild-type mice was identical to that of the “inbred” C57BL/6 strain. The phenotype of Vav2 and Vav3 heterozygous mice were similar to that of wild-type. n = 20.
(0.45 MB TIF)

Figure S2 The correlation between elevated IOP and angle changes in Vav2/Vav3-deficient mice. The IOP was measured in 7-week-old Vav2/Vav3-deficient (Vav2^−/−^Vav3^−/−^) mice (n = 20), followed by examination of the angle status by histology. While Vav2^−/−^Vav3^−/−^ mice with elevated IOP displayed histological evidence of angle closure, mice without elevated IOP showed either normal open angles or evidence of angle changes, angle closure or peripheral anterior synechiae. The mean and standard deviation of IOP in wild-type mice at 7-week-old (n = 18) were 13.7±3.12 mmHg, respectively. The 95th percentile of those IOPs using a normal curve was 18.8 mmHg. IOP over 18.8 mmHg was regarded here as elevated IOP.
(0.57 MB TIF)

Figure S3 Anti-myocilin staining of trabecular meshwork in Vav2/Vav3-deficient mice. Immunohistochemical staining of trabecular meshwork with anti-myocilin antibody in representative iridocorneal angle sections of age-matched wild-type and Vav2/Vav3-deficient (Vav2^−/−^Vav3^−/−^) 7-week-old mice with normal IOP, with either evidence of angle closure, or normal open angles similar to wild type mice. Myocilin (green-labeled), which is strongly expressed in TM cells, was regarded as a marker for TM cells. In Vav2^−/−^Vav3^−/−^ mice with angle closure, myocilin was not detected in the iridocorneal angle (indicated by arrows). Conversely, it was detected in sections from mice with normal open angles, similar to those in wild type mice. Blue fluorescence is DAPI counter staining. Scale bars, 20 um.
(2.19 MB TIF)

Figure S4 Effects of ocular hypotensives in Vav2/Vav3-deficient mice. A. Ocular hypotensives used for human glaucoma, latanoprost, a prostaglandin analogue was tested in 7-week-old Vav2/Vav3-deficient (Vav2^−/−^Vav3^−/−^) mice with elevated IOP (n = 20). The IOP was measured 3 hours before and after topical application of 3 µl of 0.01% latanoprost in a masked manner. Vehicle was used as a control. Latanoprost lowered the IOP significantly in Vav2^−/−^Vav3^−/−^ mice (26.3±5.0 mmHg versus 15.8±5.1 mmHg; n = 20), while the IOP was not altered by the vehicle alone. The latanoprost-induced reduction of IOP in Vav2^−/−^Vav3^−/−^mice was statistically significant (**P<0.01, n = 20). The data shown are representative of three independent experiments performed. Error bars represent S.D. **P<0.01 versus vehicle-treated Vav2^−/−^Vav3^−/−^ mice. B. Using three different drugs for lowering IOP, we compared the effects by percentages of elevated IOP reduction. These data are representative from three independent experiments, respectively (n = 20). Error bars represent S.D. **P<0.01 versus vehicle-treated Vav2^−/−^Vav3^−/−^ mice. C. Rho-associated protein kinase Inhibitor, Y-27632 was tested for lowering IOP on Vav2^−/−^Vav3^−/−^ mice (n = 20). Y27632 administration has no effect against Vav2^−/−^Vav3^−/−^ mice (before, 19.69±4.98 mmHg; after, 18.83±5.60 mmHg; n = 20), while Y-27632 lowered the IOP significantly in age-matched wild-type mice (13.58±2.27 mmHg versus 12.31±1.94 mmHg; n = 20. p<0.05) and the IOP was not altered by the vehicle solution (13.25±1.71 mmHg versus 13.18±3.17 mmHg; n = 20). These data are representative from four independent experiments, respectively. Error bars represent S.D. *P<0.05 versus vehicle-treated WT mice.
(0.41 MB TIF)

Figure S5 Sense probe staining for in situ hybridization experiments in ocular tissues. In situ hybridization with Vav2 and Vav3 sense probes were carried out as negative controls for the experiments. C57BL/6 mouse ocular tissue sections including the iridocorneal angle, sclera and cornea were used. With sense probes, there was no detectable signal around mouse iridocorneal angle tissues. TM; trabecular meshwork. Scl; sclera.
(4.14 MB TIF)
