# Supplementary figures and images for: VAV2 and VAV3 as Candidate Disease Genes for Spontaneous Glaucoma in Mice and Humans
Source: PLoS One. 2010 Feb 4;5(2):e9050. doi: 10.1371/journal.pone.0009050 (PMC2816215; doi:10.1371/journal.pone.0009050)

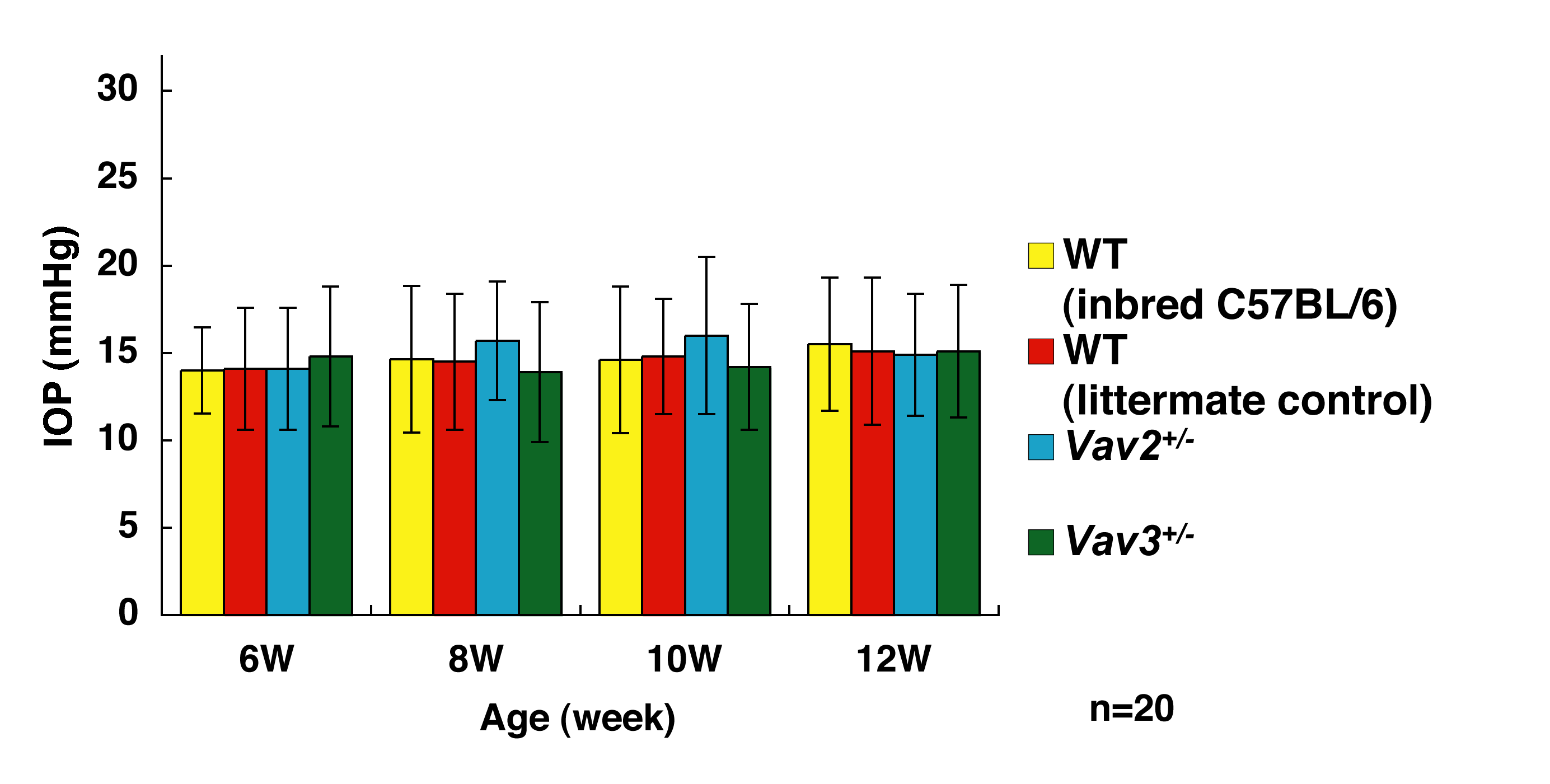

Supplement: Figure S1 — The comparison of intraocular pressures in age matched wild-type inbred C57BL/6 mice, wild-type littermate controls, and Vav2 and Vav3 heterozygous mice (Vav2+/−, and Vav3+/−). Intraocular pressures (IOPs) were measured using the TonoLab rebound tonometer for rodents from 6-week to 12-week, as described in the Methods. The phenotype of littermate wild-type mice was identical to that of the “inbred” C57BL/6 strain. The phenotype of Vav2 and Vav3 heterozygous mice were similar to that of wild-type. n = 20. (0.45 MB TIF) [file pone.0009050.s001.tif]

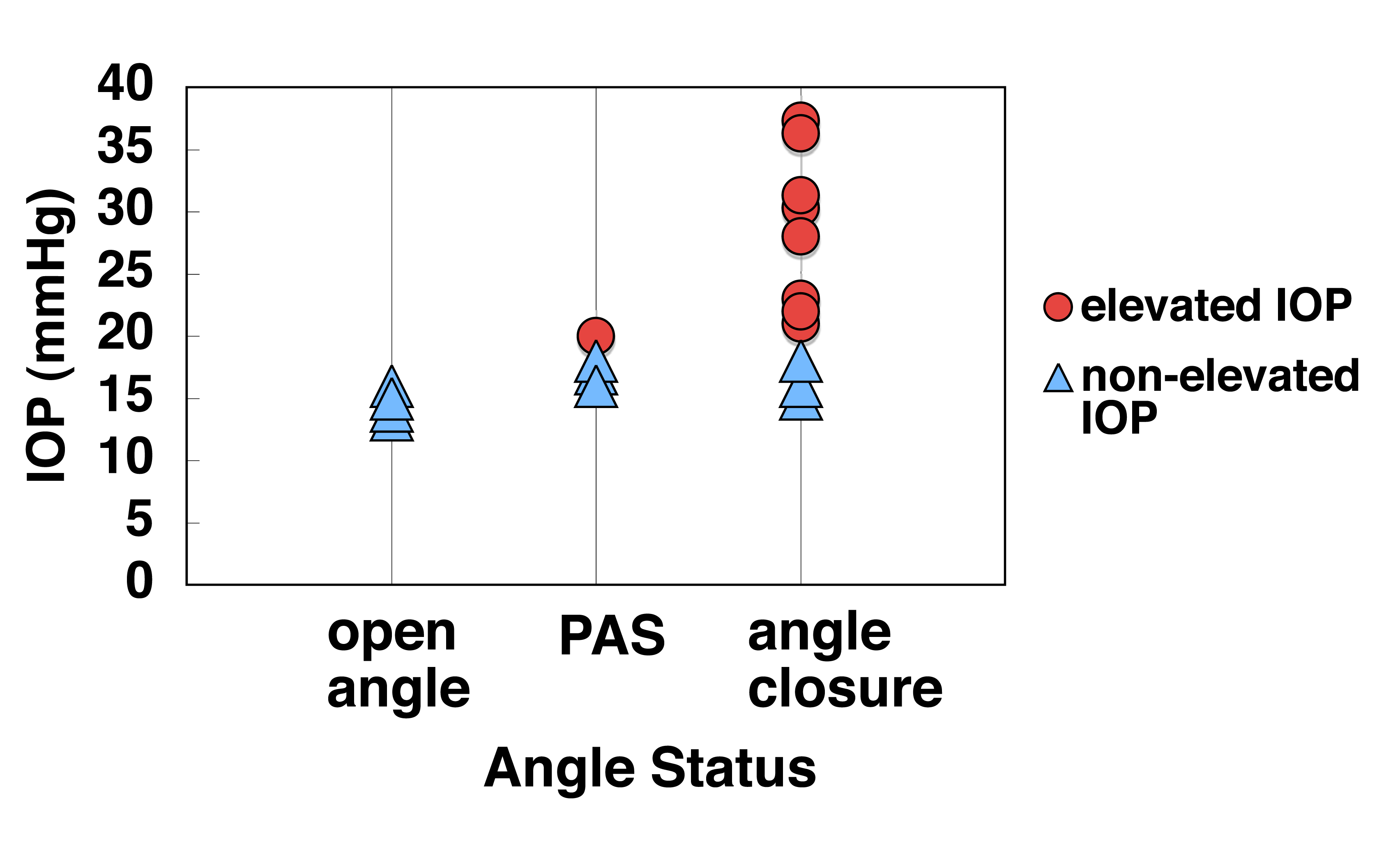

Supplement: Figure S2 — The correlation between elevated IOP and angle changes in Vav2/Vav3-deficient mice. The IOP was measured in 7-week-old Vav2/Vav3-deficient (Vav2−/−Vav3−/−) mice (n = 20), followed by examination of the angle status by histology. While Vav2−/−Vav3−/− mice with elevated IOP displayed histological evidence of angle closure, mice without elevated IOP showed either normal open angles or evidence of angle changes, angle closure or peripheral anterior synechiae. The mean and standard deviation of IOP in wild-type mice at 7-week-old (n = 18) were 13.7±3.12 mmHg, respectively. The 95th percentile of those IOPs using a normal curve was 18.8 mmHg. IOP over 18.8 mmHg was regarded here as elevated IOP. (0.57 MB TIF) [file pone.0009050.s002.tif]

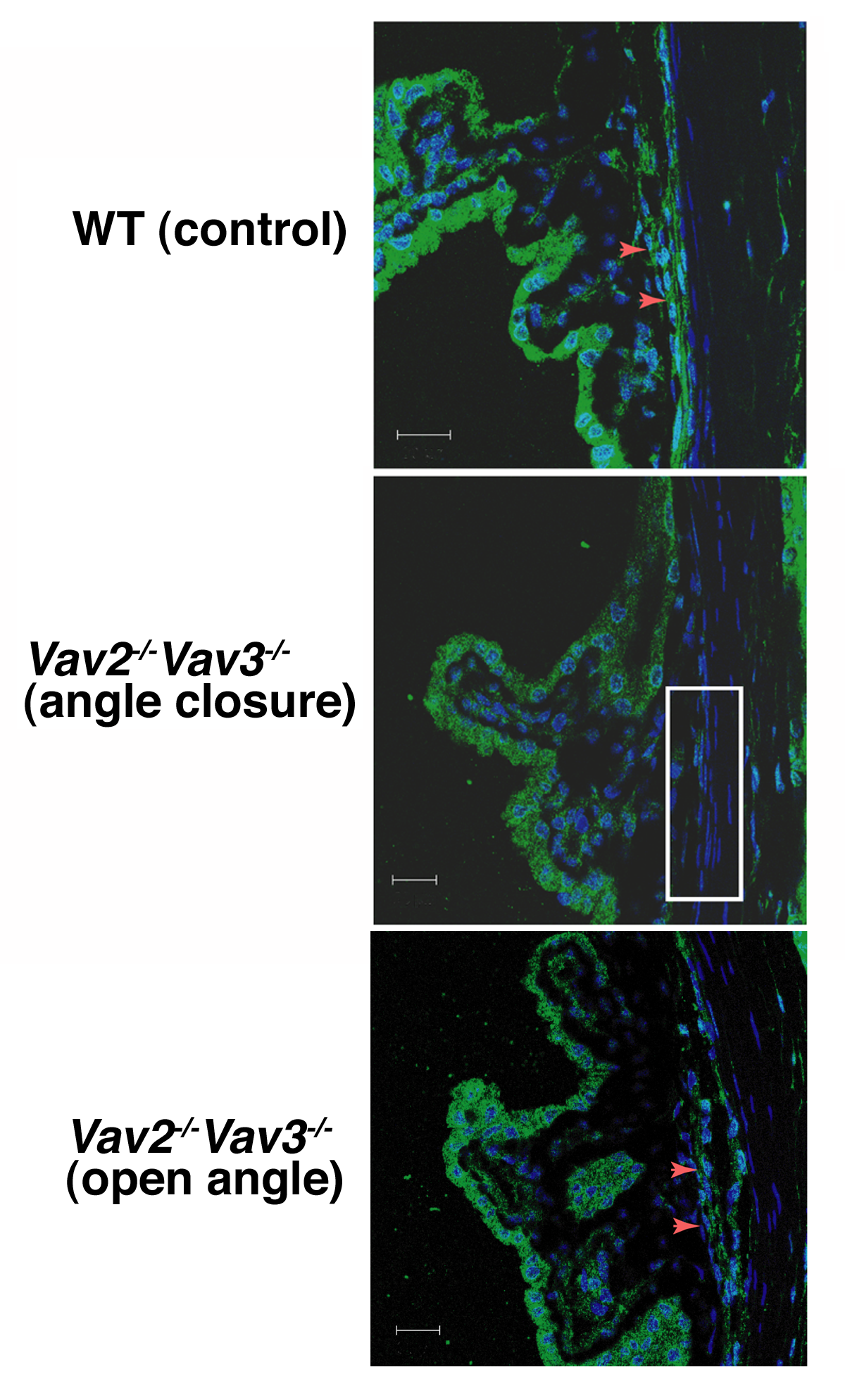

Supplement: Figure S3 — Anti-myocilin staining of trabecular meshwork in Vav2/Vav3-deficient mice. Immunohistochemical staining of trabecular meshwork with anti-myocilin antibody in representative iridocorneal angle sections of age-matched wild-type and Vav2/Vav3-deficient (Vav2−/−Vav3−/−) 7-week-old mice with normal IOP, with either evidence of angle closure, or normal open angles similar to wild type mice. Myocilin (green-labeled), which is strongly expressed in TM cells, was regarded as a marker for TM cells. In Vav2−/−Vav3−/− mice with angle closure, myocilin was not detected in the iridocorneal angle (indicated by arrows). Conversely, it was detected in sections from mice with normal open angles, similar to those in wild type mice. Blue fluorescence is DAPI counter staining. Scale bars, 20 um. (2.19 MB TIF) [file pone.0009050.s003.tif]

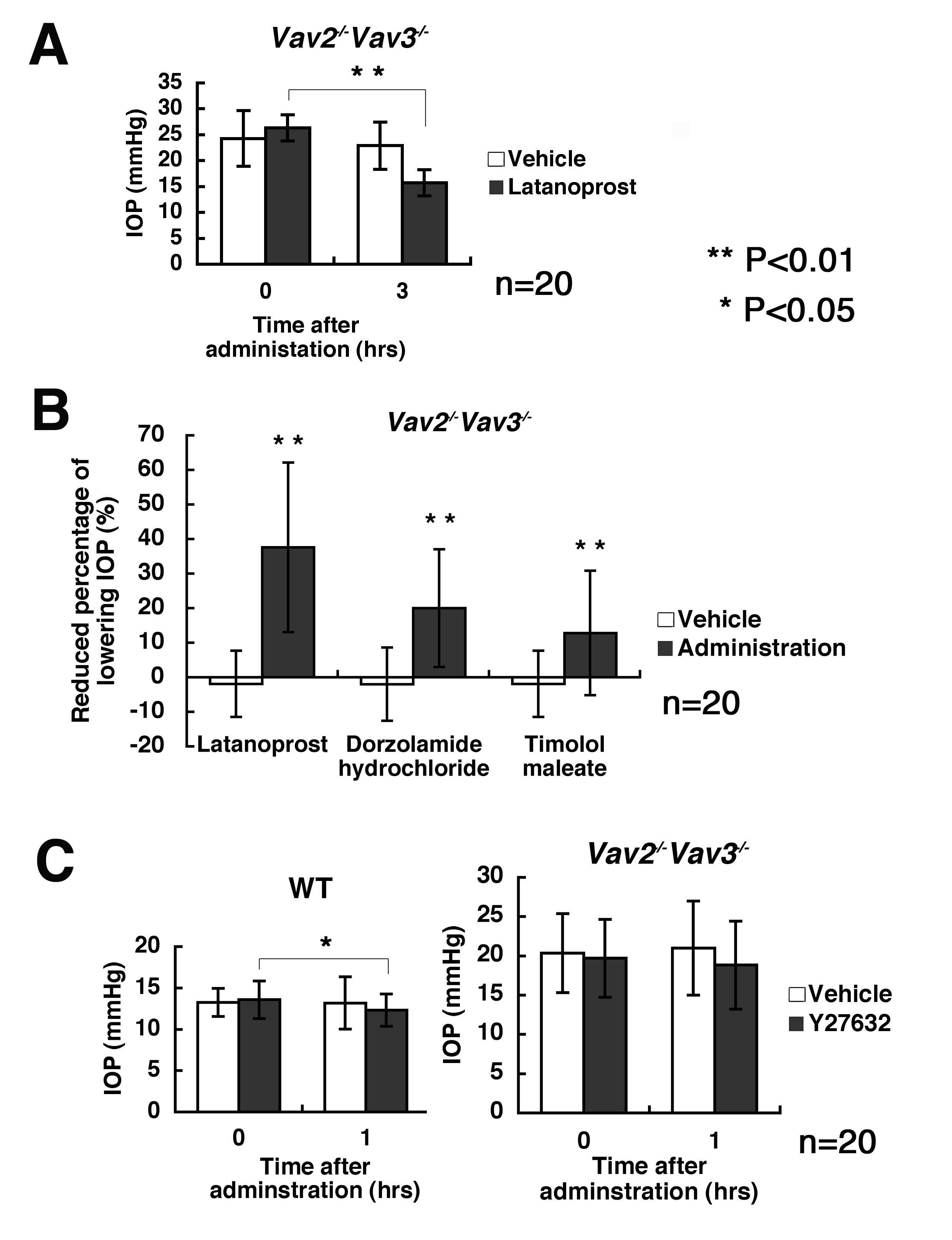

Supplement: Figure S4 — Effects of ocular hypotensives in Vav2/Vav3-deficient mice. A. Ocular hypotensives used for human glaucoma, latanoprost, a prostaglandin analogue was tested in 7-week-old Vav2/Vav3-deficient (Vav2−/−Vav3−/−) mice with elevated IOP (n = 20). The IOP was measured 3 hours before and after topical application of 3 µl of 0.01% latanoprost in a masked manner. Vehicle was used as a control. Latanoprost lowered the IOP significantly in Vav2−/−Vav3−/− mice (26.3±5.0 mmHg versus 15.8±5.1 mmHg; n = 20), while the IOP was not altered by the vehicle alone. The latanoprost-induced reduction of IOP in Vav2−/−Vav3−/−mice was statistically significant (**P<0.01, n = 20). The data shown are representative of three independent experiments performed. Error bars represent S.D. **P<0.01 versus vehicle-treated Vav2−/−Vav3−/− mice. B. Using three different drugs for lowering IOP, we compared the effects by percentages of elevated IOP reduction. These data are representative from three independent experiments, respectively (n = 20). Error bars represent S.D. **P<0.01 versus vehicle-treated Vav2−/−Vav3−/− mice. C. Rho-associated protein kinase Inhibitor, Y-27632 was tested for lowering IOP on Vav2−/−Vav3−/− mice (n = 20). Y27632 administration has no effect against Vav2−/−Vav3−/− mice (before, 19.69±4.98 mmHg; after, 18.83±5.60 mmHg; n = 20), while Y-27632 lowered the IOP significantly in age-matched wild-type mice (13.58±2.27 mmHg versus 12.31±1.94 mmHg; n = 20. p<0.05) and the IOP was not altered by the vehicle solution (13.25±1.71 mmHg versus 13.18±3.17 mmHg; n = 20). These data are representative from four independent experiments, respectively. Error bars represent S.D. *P<0.05 versus vehicle-treated WT mice. (0.41 MB TIF) [file pone.0009050.s004.tif]

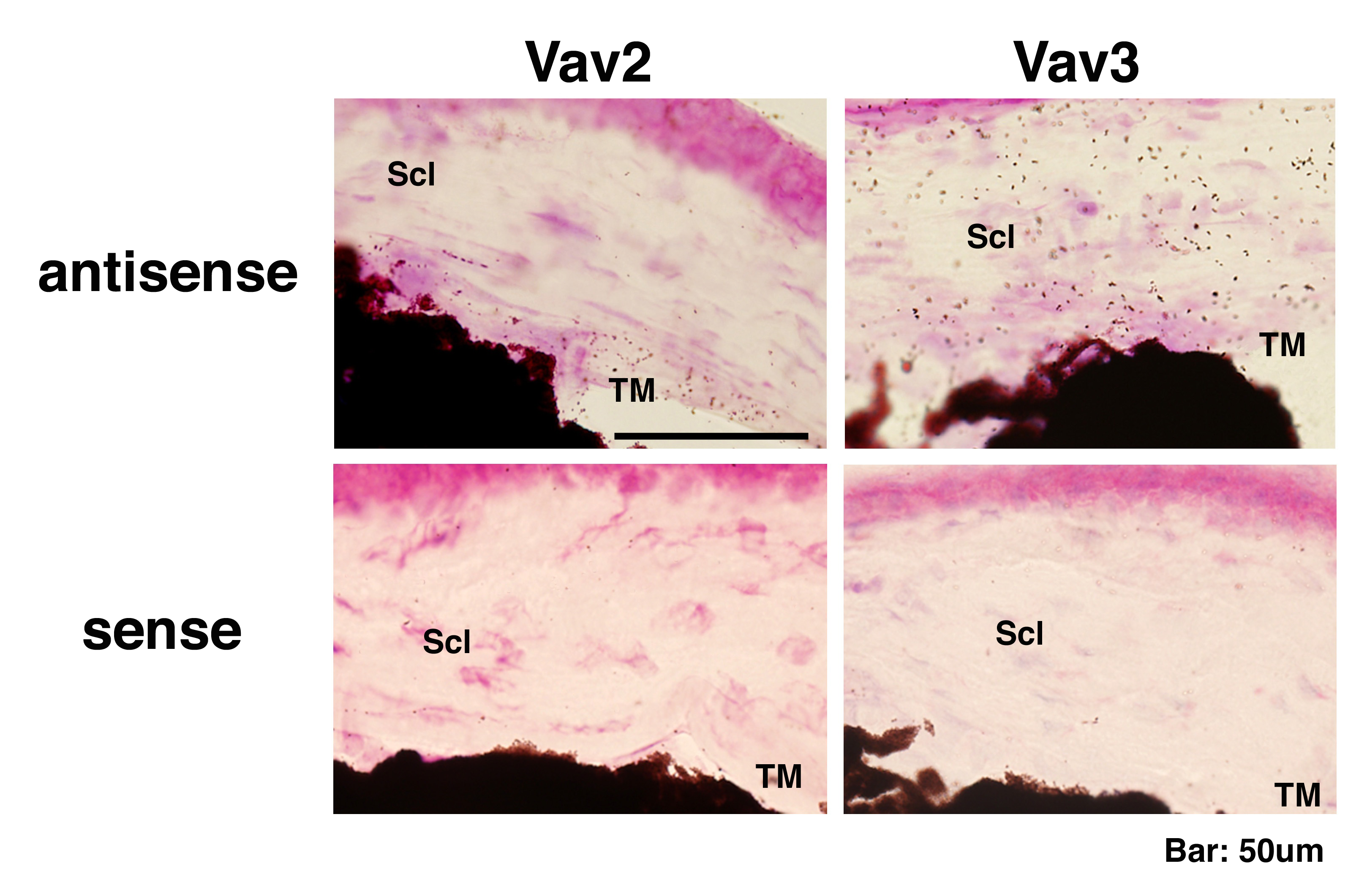

Supplement: Figure S5 — Sense probe staining for in situ hybridization experiments in ocular tissues. In situ hybridization with Vav2 and Vav3 sense probes were carried out as negative controls for the experiments. C57BL/6 mouse ocular tissue sections including the iridocorneal angle, sclera and cornea were used. With sense probes, there was no detectable signal around mouse iridocorneal angle tissues. TM; trabecular meshwork. Scl; sclera. (4.14 MB TIF) [file pone.0009050.s005.tif]
